# Age Moderates the Relationships between Family Functioning and Neck Pain/Disability

**DOI:** 10.1371/journal.pone.0153606

**Published:** 2016-04-14

**Authors:** Grażyna Guzy, Romuald Polczyk, Malwina Szpitalak, Howard Vernon

**Affiliations:** 1 Department of Physiotherapy, University of Physical Education, Cracow, Poland; 2 Institute of Psychology, Jagiellonian University, Cracow, Poland; 3 Canadian Memorial Chiropractic College, Toronto, Canada; Federal University of Rio de Janeiro, BRAZIL

## Abstract

This cross-sectional clinical study was designed to explore the relationships between family functioning, coping styles, and neck pain and neck disability. It was hypothesized that better family functioning and more effective coping styles would be associated with less pain and pain-related disability. It also was hypothesized that these relationships would be stronger in older people because they have fewer resources, more limited coping styles, and may depend more on their family for support. In this study, 88 women with chronic non-traumatic neck pain completed the Family Assessment Measure (FAM), Coping Inventory for Stressful Situations (CISS), Neck Disability Index (NDI), and a Visual-Analogue Scale (VAS) measuring the subjective intensity of neck pain. Zero-order and partial correlations and hierarchical stepwise regression were performed. CISS was not correlated with the NDI orVAS. Good family functioning was correlated with lower NDI and VAS scores. Age was found to moderate the relationship between the FAM and both NDI and VAS. This relationship was significant and positive in older patients, but non-significant in younger patients. It was concluded that better family functioning is associated with lower neck disability and pain intensity, especially in the case of older women suffering from non-traumatic neck pain.

## Introduction

Neck pain is commonly reported. It tends to be chronic and results in prolonged disability [1–3]. It is estimated that chronic neck pain, with various etiologies, affects between 4.8% to 79.5% of the general population each year [2,3]. As with any kind of chronic pain, it affects a wide range of areas of human life, and as such it poses a serious challenge to socio-economic systems [2,3]. We explored several aspects of the psychological functioning of patients suffering from neck pain in this study. Specifically, we analyzed the association of pain intensity and disability, as dependent variables, with coping with stress and family functioning, as independent variables (or predictors). We also analyzed the moderating effect of age on these associations.

Research on the association of psychological factors with neck pain and neck disability is relatively common. To date, this research has established that self-rated disability is positively related to depression [4], pain catastrophising [5–7], strategies of coping with pain [7], causal beliefs [5], fear of movement [6,8,9], and cervical non-organic signs [9] in whiplash sufferers. In samples of patients with mixed neck-pain (whiplash and non-whiplash neck problems), self-rated disability has been found to be correlated positively with depression, somatization [10], and the Mental Component Summary of the SF-36 [11]. Self-rated disability has been found to be strongly associated with fear of movement among pain patients who did not sufferer whiplash [12].

The importance of chronic pain has been strongly emphasized in the context of family functioning [13–16]. Lewandowsky [16] stressed that the experiences of the patient and his/her family may differ in many ways, starting with the simple fact that it is the patient who feels the pain, not the family.

The patient-family relationship can be bi-directional in that the pain patient may affect family functioning, while variables related to the family may affect various aspects of the patient’s life. Most existing studies concentrate on the first of these relationships; the patient’s effect on the family. It has been noted, among other things, that pain reduces full time employment and increases the amount of time spent doing housekeeping and household maintenance [17]. Thomas et al. [18] suggested that headache and backache, together with life-stage issues, may contribute to family dysfunction among students and their parents. On the other hand, serious conflicts in the family may aggravate chronic pain [19]. Family communication becomes centered on illness; the social life of family members suffer; pain becomes the factor that binds the family together, etc. [16].

In the present research, we were interested mainly in how family functioning affects pain and disability. Therefore, family functioning was the independent variable, or predictor, in our analyses, and neck pain and pain-related disability were the dependent variables. It should also be noted that, to our knowledge, there have been no studies on the family dynamics of patients with chronic neck pain. We hypothesized that better family functioning would result in less pain and disability. This could occur for quite simple reasons. For example, the members of the family may help patients with everyday activities, thus, enabling them to rest and concentrate more on treatment. Also, it is possible that a supportive family may encourage patients to start treatment.

Coping style would seem to be an obvious predictor of pain: the better and the more adaptive the coping style, the less the pain should be. People struggling with prolonged pain naturally have to develop strategies to cope with it [7]. However, in light of the research literature, the picture is not that clear. Some research [20] has found little evidence that either the duration or intensity of pain were strongly related to general coping styles. Avoidance-oriented coping, as measured by the Coping Inventory for Stressful Situations [21], has been found to be negatively correlated with pain intensity among patients visiting a pain clinic, which may mean that avoidance may help to reduce pain. In contrast, Hart et al. [22] found that coping through denial led to greater pain severity among HIV/AIDS patients. Some authors [23,24] have suggested that, among general pain patients, factors related to acceptance may be more important for pain and disability than factors that are related to coping. However, Esteve et al. [25] found that both acceptance and coping were important for various aspects of dealing with pain. In sum, it seems that the effect of coping strategies on pain and disability vary. Despite the inconsistencies in the existing results, we believe that the hypothesis that there is a negative relationship between the quality of coping styles and pain and disability is tenable.

In addition, we investigated whether age acted as a moderating variable. We were unable to locate any study that examined our particular question: whether age moderates the effect of family functioning and coping styles on neck pain and neck disability. There is substantial research on the association of age with neck pain and the preponderance of these studies indicate that age is connected with both the prevalence and incidence of neck pain [1–3]. Also, there are insightful analyses that relate age to coping with illness. For example, after an extensive analysis of the research literature, Berg and Upchurch [26] presented a developmental-contextual model, which suggested that the way patients and their spouses cope with an illness varies across the lifespan. However, we have found no studies on the moderating effect of age on the relationship of family functioning with coping and pain and disability.

Despite the lack of relevant research, we believe the hypothesis that age moderates the association of family functioning and coping styles on pain is plausible. Moderation, in this context, means that family variables and coping styles influence pain and disability differently in people of different ages. It is possible that the relationship between the quality of family life and experienced pain is not as strong among younger people as it is among elderly people. This could be because young people may have a wider range of possible resources (both individual and social) at their disposal, and, thus, they may not be so dependent on family support. In contrast, older people may be more dependent on their spouse (or adult children); thus, there may be a stronger relationship between their quality of family life and pain and disability.

We also were unable to find research on the relationship between coping styles and pain in which age was studied as a moderator. Nevertheless, a similar hypothesis may be tenable as in the case of family functioning: younger people may have sufficient available resources that may make them less dependent on the quality of their coping styles. Elderly people, on the other hand, may have fewer available resources, which makes them more dependent on the quality of their coping styles.

Thus, this study tested the following hypotheses:

1. The quality of family functioning is negatively related to neck pain and neck disability.
2. Effective coping styles are negatively related to neck pain and neck disability.
3. The relationship between the quality of family functioning and pain intensity and disability is stronger in younger people than in older people.
4. The relationship between coping styles and pain intensity and disability is stronger in younger people than in older people.

We restricted the sample to women in this study. This was done to avoid complications stemming from the fact that the gender roles of women and men differ in the family [27], and the relationships under study may also differ by gender. These potential gender differences would require appropriate interaction analyses that included gender as a moderator. This would require a larger sample size, which was not possible in the present research because of constraints resulting from the fact that the participants were recruited while undergoing therapy. A factor that favoured limiting the sample to females is that women seem to be more vulnerable to neck pain, and less likely to have complete relief from pain and disability than men are [1–3].

## Materials and Methods

### Subjects

The study was conducted in 2009–2010 in an outpatient clinic in Cracow, Poland. This study was part of a larger study on the psychometric properties of the Polish NDI [28]. The study was approved by the Institute of Psychology, Jagiellonian University Research Ethics Board. Written consent was provided by all the participants. The inclusion criteria for the current study were: being female; having chronic (> seven weeks), mechanical, non-specific neck pain in the area of the neck, radiating to the top of the shoulders and head; and being age 18 years or older. The exclusion criteria were: having symptoms in the upper extremities, neurologic deficits, severe coexisting neurological, rheumatic, vascular disease, malignancies, advanced diabetes, cardiac and kidney failure, mental disorders, being pregnant, and unsystematic participation in the treatment program.

### Instruments

The Neck Disability Index [28,29] is a self-administered questionnaire that includes 10 items on pain and pain-related limitations in daily activities. The total score on the NDI was expressed as a score out of 50 in the present study. The reliability of the original version of the NDI, as measured by Cronbach’s alpha, was .82. It was .81 in the present study. Guzy et al. [28] reported that the validity of the Polish version of the NDI was satisfactory.

The Visual-Analogue Scale (VAS) is a reliable and simple self-rated index of the intensity of neck pain [30]. We used a 10-cm straight line in the present study that extended from ‘no pain’ to ‘pain as bad as it could possibly be’.

The Coping Inventory for Stressful Situations (CISS) measures coping styles [21,31]. It includes three dimensions of coping styles: Task Oriented (focusing on solving problems), Emotion Oriented (emotional reactions), and Avoidance Oriented, which includes two subscales: Involvement in Other Tasks and Social Contacts. Strelau et al.’sstudy of the Polish version [31] reported the following Cronbach alphas for the three scales and the two subscales, for students: .86, .82, .75, .73, .75, respectively. The validity of CISS was reported to be satisfactory. The Cronbach’s alphas for the five subscales were .87, .88, .77, .75, and .82, respectively, in the present study.

The Family Assessment Measure (FAM) assesses the functioning of a family by family members [27,32]. The Polish version [33] of the FAM is an adaptation of the Family Assessment Measure (FAM III), developed by Cierpka and Frevert [34], which in turn, is a modification of the FAM III of Skinner et al. [32]. It contains three components: the Family Questionnaire (FQ), describing the functioning of a family as a whole, the Self-Estimating Questionnaire (SE), focusing on an individual member’s own functioning in the family, and the Dyadic Relationship Scale (DR), examining how a family member views his or her relationship with another family member (in this study, with the child of the participant). Each of the three versions assesses seven dimensions: Task Accomplishment, Role Performance, Communication, Emotionality, Affective Involvement, Control, and Values and Norms. The higher the scores, the worse the functioning of the family is in a given area. The Family Questionnaire also includes two additional control scales measuring Social Expectations and Defence, in which the higher the scores, the better functioning is. Beauvale et al. [33] reported extremely diverse Cronbach alphas for the FAM, depending on the sample, which ranged from .36 to .81. The validity of the FAM was satisfactory. Most of the subscales of the FAM in the present study showed satisfactory reliability, ranging from .69 to .87, with the following exceptions: the FQ’s Affective Involvement (alpha = .50) and Control (.59); the SE’s Control (.46); and the DR’s Emotionality (.46).

### Procedure

All the data analyzed in this article were obtained from the baseline measurements. Patients were examined by a medical doctor upon entering the study. The diagnosis was based on an interview, a physical examination, and imaging tests. Then, all the participants were informed about the aim of the project, its procedures, anonymity, and voluntary participation, and were asked to complete the NDI, CISS, and FAM; the level of neck pain was determined using the VAS. The study was conducted in collaboration with psychologists, and the measurements were performed by experienced physiotherapists who were blinded to the study.

### Statistical procedures

Zero-order and partial correlations (controlling for age) and a combination of hierarchical and stepwise regression models were performed, in which age was entered in the first model, and the remaining predictors (related to family functioning and coping styles) were entered into the subsequent models using the stepwise method to analyze moderation effects. The program PROCESS was used to analyze moderation effects [35].

## Results

In sum, eighty-eight women took part in the study. Their mean age was 54.5 (*SD* = 14.8; range 22–87). The mean of the NDI was 15.69 (*SD* = 5.50; range 7–33) and the mean of the VAS was 47.96 (*SD* = 12.02; range 17–82). According to the criteria suggested by Vernon and Mior [28], the sample may be classified as follows: mild (43; 48.9%), moderate (41; 46.6%), and severe disability (4; 4.5%). The categories “none” and “complete” were not present in the sample. A lower level of disability is typical forthis subgroup of patients suffering from non-traumatic neck pain and it is similar to that observed by Vos et al. [36].

First, correlations were performed between the NDI and VAS and variables related to family functioning and coping with stress. The correlations of all the variables with the age of the participants are included in Table 1.

**Table 1:** Zero-order and partial correlations among the VAS, NDI and age, and family functioning and coping strategies.

		Zero-order correlations			Partial correlationscontrolling for age	
Questionnaires	Subscales	Age	VAS	NDI	VAS	NDI
**VAS**	-	-.07	-	-	-	-
**NDI**	-	.49**	.49**	-	.61**	-
**CISS**	Task Oriented	-.29*	.11	-.13	.09	.02
**CISS**	Emotion Oriented	.27*	.04	.08	.07	-.06
**CISS**	Avoidance Oriented	-.04	-.01	-.11	-.01	-.10
**CISS**	Involvement in other task	.16	.00	-.04	.01	-.13
**CISS**	Social contacts	-.30**	.04	-.10	.02	.05
**FQ**	Task Accomplishment	.25*	.36**	.30**	.39**	.21*
**FQ**	Role Performance	.14	.40**	.28*	.41**	.24*
**FQ**	Communication	.16	.38**	.26*	.39**	.21
**FQ**	Emotionality	.22*	.29*	.26*	.32**	.18
**FQ**	Affective Involvement	.26*	.39**	.31**	.43**	.22*
**FQ**	Control	.28**	.11	.21*	.13	.09
**FQ**	Values and Norms	.17	.22	.23*	.24*	.17
**FQ**	Social Expectations	-.20	-.34**	-.31**	-.36**	-.24*
**FQ**	Defence	-.11	-.30**	-.13	-.31**	-.09
**SE**	Task Accomplishment	.33**	.17	.21	.21	.06
**SE**	Role Performance	.14	.23*	.15	.24*	.10
**SE**	Communication	.14	.28*	.24*	.29**	.20
**SE**	Emotionality	.25*	.21	.23*	.24*	.13
**SE**	Affective Involvement	.21	.26*	.28**	.28*	.21
**SE**	Control	.17	.05	.23*	.06	.17
**SE**	Values and Norms	.27*	.18	.30**	.21	.20
**DR**	Task Accomplishment	-.07	.24*	.12	.24	.17
**DR**	Role Performance	-.10	.24	.03	.24	.09
**DR**	Communication	.03	.18	.11	.19	.11
**DR**	Emotionality	.14	.09	.18	.11	.13
**DR**	Affective Involvement	.20	.13	.18	.15	.10
**DR**	Control	.09	.20	.08	.21	.04
**DR**	Values and Norms	.15	.27*	.18	.29*	.12

VAS: Visual-Analogue Scale measuring neck pain; NDI: Neck Disability Index; FQ: Family Questionnaire; SE: Self-Estimating Questionnaire; DR: Diadic Relationship Scale

* *p*< .05;

** *p*< .01

The correlation between age and the NDI was significant and positive, in contrast to the VAS. Age had a significant positivecorrelationwith Emotion Oriented style of coping, and a negative correlation with Task Oriented and Social Contacts. Age also had significant positive correlations with the FQ’s Task Accomplishment, Emotionality, Affective Involvement, and Control subscales and the SE’s Task Accomplishment, and Emotionality subscales.

Neither the VAS nor the NDI were significantly correlated with any of the coping styles. However, both the VAS and the NDI were positively correlated with the FQ’s Task Accomplishment, Role Performance, Communication, Emotionality, Affective Involvement subscales, the SE’s Communication andAffective Involvement subscales, and negatively correlated with the FQ’s Social Expectations subscale. Apart from this, the VAS was positively correlated with the SE’s Role Performance subscale and the DR’s Task Accomplishment and Values and Norms subscales, and negatively correlated with the FQ’s Defence subscale. The NDI was positively correlated (apart from the above-mentioned correlations) with the FQ’s Control and Values and Norms subscales, and the SE’s Emotionality, Control, and Values and Norms subscales. The correlations were, at most, moderate, according to the standards proposed by Taylor [37].

Controlling for age did not have any effect on the correlations with regard to the CISS. Most of the correlations with the FQ remained significant after controlling for age. In the case of the SE, some correlations became non-significant when age was controlled; namely, those between the NDI and the SE Communication, Emotionality, Affective Involvement, Control, and Values and Norms subscales.

Next, hierarchical stepwise regressions were performed. To reduce the number of predictors, which would be relatively large in relation to the sample size, the coping styles were dropped from these analyses, as they were not correlated with the VAS or the NDI in bivariate correlations or the partial correlations, which controlled for age. Age was included in the first block, and the remaining predictors were entered in the second block of variables by means of stepwise elimination. Six analyses were performed, each with three groups of predictors: the subscales of the FQ, SE, and DR (Table 2).

**Table 2:** Multiple hierarchical-stepwise regression models with VAS and NDI as dependent variables and family functioning as predictors.

Dependent variable	Group of predictors	*R*^*2*^_*adj*_	*F*	*p*_*M*_	Predictors in the final model	*Beta*	*r*^*2*^_*semi*_	*t*	*p*_*P*_
**VAS**	Subscales of the FQ	.15	7.44	.001	FQ—Affective Involvement	0.43	.17	3.83	< .001
**VAS**	Subscales of the SE	.07	3.86	.025	SE—Communication	0.31	.09	2.69	.009
**VAS**	Subscales of the DR	.05	2.67	.077	-	-	-	-	-
**NDI**	Subscales of the FQ	.28	16.14	< .001	Age	0.40	.15	3.99	< .001
**NDI**					FQ—Task Accomplishment	0.28	.07	2.77	.007
**NDI**	Subscales of the SE	.21	22.47	< .001	Age	0.47	.22	4.74	< .001
**NDI**					SE—Affective Involvement	0.19	.04	1.94	.056
**NDI**	Subscales of the DR	.22	21.03	< .001	Age	0.48	.23	4.59	< .001

VAS: Visual-Analogue Scale measuring neck pain; NDI: Neck Disability Index; CISS: Coping Inventory for Stressful Situations; FQ: Family Questionnaire; SE: Self-Estimating Questionnaire; DR: Diadic Relationship Scale. Please note: in all analyses, age was entered as the first step. Please note: the constants were omitted in the table. *r*^*2*^_*semi*_: squared semi-partial correlations (an index of the proportion of variance explained). *p*_*M*_: *p* value for the entire model. *p*_p_: *p* value for the predictor.

Table 2 shows the predictors of the dependent variables VAS and NDI. Starting with the VAS, the FQ’s Affective Involvement subscale proved to be a significant predictor, explaining about 17% of its variance. The SE Communication subscale also was a significant predictor in VAS, explaining about 9% its variance. No DR subscale was a significant predictor of VAS. Age was not retained in the final equation of any of the three analyses of the VAS (Table 2).

As for the NDI, the FQ subscale Task Accomplishment proved to be a significant predictor, explaining about 7% of the variance in the NDI. Age was also present in the final model, explaining 15% of the variance in the NDI. Among the SE subscales, Affective Involvement was barely significant (4% of the variance explained). Age also was significant, accounting for 22% of the variance in the NDI. Finally, none of the DR subscales were retained in the final models, but age accounted for 23% of the variance in the NDI (Table 2).

Next, the moderator analyses were completed to assess whether age was a moderator of the relationship between coping and family functioning on one side, and VAS and the NDI on the other side. The significant results are presented in Table 3.

**Table 3:** Results of the regression analyses testing the interaction between age and the predictors (dependent variables: NDI and VAS).

Dependent variable	Predictor	*Beta*	*SE*	*T*	*p*	*β*_*L*_	*β*_*M*_	*β*_*H*_
**NDI**	FQ—Role Performance	.21	.10	2.12	.038	-.08	.15	.38**
**NDI**	FQ—Communication	.22	.10	2.17	.033	-.13	.09	.31**
**NDI**	FQ—Emotionality	.23	.09	2.43	.017	-.12	.11	.34**
**NDI**	FQ—Affective Involvement	.23	.09	2.39	.019	-.11	.13	.37*
**NDI**	FQ—Social Expectations	-.25	.09	-2.77	.007	.10	-.16,	-.41**
**NDI**	SE—Role Performance	.23	.10	2.40	.019	-.12	.11	.35*
**NDI**	SE—Communication	.30	.09	3.25	.002	-.22	.11	.43**
**NDI**	SE—Emotionality	.30	.10	2.94	.004	-.32	-.01	.31**
**NDI**	SE—Control	.21	.10	2.16	.034	-.08,	.13	.35*
**NDI**	DR—Task Accomplishment	.21	.10	2.02	.047	-.08,	.12	.32*
**NDI**	DR—Communication	.28	.10	2.77	.007	-.25	.05,	.35*
**NDI**	DR—Emotionality	.37	.10	3.78	< .001	-.31*	.10	.50**
**NDI**	DR—Affective Involvement	.30	.10	2.88	.005	-.28	.00	.28*
**NDI**	DR—Values and Norms	.20	.10	1.99	.051	-.15	.07	.29*
**VAS**	FQ—Values and Norms	.29	.11	2.64	.010	-.08	.19	.46**
**VAS**	SE—Communication	.25	.11	2.25	.027	-.03	.23*	.50**
**VAS**	SE—Emotionality	.26	.12	2.12	.037	-.13	.14	.41**
**VAS**	DR—Communication	.27	.12	2.20	.032	-.15	.14	.43**
**VAS**	DR—Emotionality	.28	.13	2.12	.038	-.20	.10	.39*
**VAS**	DR—Affective Involvement	.10	.05	2.20	.031	-.20	.06	.33*

VAS: Visual-Analogue Scale measuring neck pain; NDI: Neck Disability Index; FQ: Family Questionnaire; SE: Self-Estimating Questionnaire; DR: Diadic Relationship Scale. *β*_*L*_, *β*_*M*_, *β*_*H*_: beta coefficients for the relationship between a given predictor and the dependent variable, respectively, at low (1 *SD* below the mean), medium (mean), and high (1 SD above the mean) values of age. Only significant results are included in the table.

* *p*< .05;

** *p*< .01

The pattern of results obtained was strikingly consistent across the different predictors and both dependent variables (NDI and VAS), with significant associations between the predictors and the dependent variables in the older participants, but not the younger ones. The association of the following predictors of the NDI interacted with age and were significant predictors of the NDI in older participants: (a) the FQ’s Role Performance, Communication, Emotionality, Affective Involvement, and Social Expectations subscales; (b) the SE’s Role Performance, Communication, Emotionality, and Control subscales; and (c) the DR’sTask Accomplishment, Communication, Emotionality, Affective Involvement, and Values and Norms subscales (Table 3).

Significant moderation effects were also found when the VAS was the dependent variable, in which the following predictors interacted with age: (a) the FQ’s Values and Norms subscale; (b) the SE’s Communication and Emotionality subscales; and (c) the DR’s Communication, Emotionality, and Affective Involvement subscales.

As seen in Table 3, there was one analysis in which age and a family-related variable had a negative interaction effect on NDI: DR Emotionality. This means that the DR’s Emotionality subscale was *negatively* associated with the NDI in younger persons, but positively associated with the NDI in older persons. In both sets of analyses, only family-related variables proved to be involved in the moderating effects, not coping styles (Table 3).

## Discussion

The main aim of this study was to analyze the relationship between family functioning and coping styles, on the one hand (the independent variables), and neck pain and neck disability on the other (the dependent variables). We also sought to determine whether this relationship is moderated by age; that is, whether this relationship is different at various ages.

In sum, the relationships between family functioning and neck pain and neck disability were found to be statistically significant, with the vast majority of the scales of the FAM correlating with the VAS and NDI. The direction of the obtained correlations indicated that better family functioning is associated with less subjectively experienced pain and disability. This supports our first hypothesis that the quality of family life influences the experience of pain and disability, although it must be stressed that these results are based on correlational data, so one cannot infer the direction of causation.

The number of significant effects found in our analysis was much larger in the correlational analyses than in the multiple regression analyses. This may be due to the fact that the intercorrelations among the subscales of the FAM were quite high (see S1 Table), which may mean that they all assess similar dimensions. In light of this, the results of the regression analyses should be treated with some caution. The variables that were found to be significant in the regression analyses (Affective Involvement, Communication, Task Accomplishment, and Affective Involvement) may be interpreted as the most important predictors of pain and disability, as they were significant after all the other variables were controlled. However, the predictors that were not significant in the regression analyses, but were significant in the bivariate correlation analyses, may also be important.

Given the lack of existing research in this area, we can only speculate about the reason for the negative correlation between family function and neck pain/disability. One currently and widely accepted mechanism—fear-avoidance beliefs—may help to explain these results [38,39]. The Fear-Avoidance Model postulates that pain-related avoidance of physical activity may lead to persistent pain. Therefore, it maybe that, in the case of older women, good family functioning has the effect of encouraging neck-pain patients to have less fear of movement to safely participate in family activities; consequently, as pain depends to some extent on fear, the patient may experience less pain as well. The same may be true in the case of neck disability [6,12]. Future studies should include assessment of fear-avoidance beliefs to confirm this hypothesis.

A second mechanism operating either separately or in conjunction with fear-avoidance beliefs may be the presence of depression. Depression has been found to be positively associated with higher levels of neck disability [4,6]. Poorer family functioning may promote depression among older female neck-pain sufferers, thus, promoting higher pain and disability scores. Future studies should include the assessment of depression to confirm this possibility.

The results of the moderation analyses showed that the relationship between family functioning and pain and disability was significant in older women, but not in younger women. This finding is in line with the hypothesis that elderly people may be more dependent than younger people on the support of their families. Younger neck-pain sufferers may be able to seek help and obtain sufficient support regardless of whether such help is available in the family context, whereas older sufferers may be more dependent on family support. Thus, in the case of older people, aspects of the quality of family functioning, such as proper role performance, communication, emotionality, affective involvement, and social expectations, may help them handle their pain and disability.

When interpreting these results, it should be borne in mind that only women participated in this study. It is possible that women are more dependent on the quality of family life than men are [40].

Regardless of the actual mechanism underlying the moderating effect on the relationship between family functioning and neck-related problems, these results are important in that the population is aging in most countries [41,42]. In light of the results of the moderation analyses presented in this article, health professionals should take into account the family situation of an elderly female patient, as it is possible that family problems may contribute to health problems.

It was somewhat surprising that coping styles were not significantly related to either neck pain or the disability related to neck pain (neither as the main effect, nor in the interaction effect with age). Many studies have shown that coping is an important predictor of pain [43]. However, as elaborated in the Introduction, the relationship between coping and pain is a complicated matter, and variables, such as pain acceptance, may be more important [23].

With regard to the specific topic of neck area, coping styles are often found to be unrelated, or at best weakly related, to pain. For example, Hurwitz et al.’s [44] analysis of the outcomes of a six-month therapy program showed that certain coping strategies (self-assurance) were positively related to the outcomes of chiropractic treatment of neck pain. Nieto et al. [7] only obtained some relationships between coping and “asking for assistance” and “disability” in their regression analyses, and some correlations in their bivariate analyses. In sum, it seems that, in the light of the present results and those in the literature, coping styles are not consistently related to neck pain and neck disability. However, our results should not be generalized to patients with other kinds of pain.

It may be that the lack of significant effects of coping styles is related to the fact the present study used general coping styles related to coping with stress (as measured by the CISS), whereas most research of this kind has used coping strategies that are more directly related to coping with pain [45].

Finally, it is worth mentioning that the present study found positive correlations between age and neck disability (as measured by the NDI), but not age and neck pain (VAS). This is somewhat surprising, as most studies of this kind have consistently found age to be unrelated to the NDI, in the case of both traumatic injury [5,7–9], and non-traumatic patients [46], as well as mixed samples [47,48]. In some research, age was a significant predictor only at some steps of the multiple regression analyses [6,8,9]. It is possible that some cross-cultural economic differences may be important here, as the cited research was conducted mostly in western countries. It is possible that in Poland, which is relatively less wealthy than many western countries [49], and where medical treatment may not be equivalent to western standards, older people may experience poorer health [50,51].

## Conclusion

Poorer family functioning may be connected with higher neck-pain disability and intensity, especially in the case of older women suffering from non-traumatic neck pain. Family functioning should be investigated in all neck-pain patients, especially those patients in their later years of life.

## Supporting Information

S1 Table Intercorrelations among the subscales of the Family Questionnaire. (DOCX)

S2 Table Intercorrelations among the subscales of the Self-Estimating Questionnaire. (DOCX)

S3 Table Intercorrelations among the subscales of the Diadic Relationship Scale. (DOCX)

S4 Table Results of regression analyses testing for interactions between age and predictors (dependent variable: Neck Disability Index)—non-significant results. (DOCX)

S5 Table Results of regression analyses testing for interactions between age and predictors (dependent variable: Visual Analogue Scale (pain))—non-significant results. (DOCX)

S6 Table Multiple hierarchical-stepwise regressions for Neck Disability Index as the dependent variable and family functioning (Family Questionnaire) as predictors—non-significant results. (DOCX)

S7 Table Multiple hierarchical-stepwise regressions for Neck Disability Index as the dependent variable and family functioning (Self-Estimating Questionnaire) as predictors—non-significant results. (DOCX)

S8 Table Multiple hierarchical-stepwise regressions for Neck Disability Index as the dependent variable and family functioning (Diadic Relationship Scale) as predictors—non-significant results. (DOCX)

S9 Table Multiple hierarchical-stepwise regressions for Neck Disability Index as the dependent variable and coping styles as predictors—non-significant results. (DOCX)

S10 Table Multiple hierarchical-stepwise regressions for the Visual-Analogue Scale (pain) as the dependent variable and family functioning (Family Questionnaire) as predictors—non-significant results. (DOCX)

S11 Table Multiple hierarchical-stepwise regressions for Visual-Analogue Scale (pain) as the dependent variable and family functioning (Self-Estimating Questionnaire) as predictors—non-significant results. (DOCX)

S12 Table Multiple hierarchical-stepwise regressions for Visual-Analogue Scale (pain) as the dependent variable and family functioning (Diadic Relationship Scale) as predictors—non-significant results. (DOCX)

S13 Table Multiple hierarchical-stepwise regressions for Visual-Analogue Scale (pain) as the dependent variable and coping styles (CISS) as predictors—non-significant results. (DOCX)
