# Supplementary material for: Age Moderates the Relationships between Family Functioning and Neck Pain/Disability
Source: PLoS One. 2016 Apr 14;11(4):e0153606. doi: 10.1371/journal.pone.0153606 (PMC4831820; doi:10.1371/journal.pone.0153606)
Supplement: S1 Table — (DOCX) [file pone.0153606.s001.docx]

**S1 Table. Intercorrelations among the subscales of the Family Questionnaire.**

|  | **FQ - Task Accomplishment** | **FQ - Role Performance** | **FQ - Communication** | **FQ - Emotionality** | **FQ - Affective Involvement** | **FQ - Control** | **FQ - Values and Norms** | **FQ - Social Expectation** |
| --- | --- | --- | --- | --- | --- | --- | --- | --- |
| **FQ - Role Performance** | .85** |  |  |  |  |  |  |  |
| **FQ - Communication** | .86** | .84** |  |  |  |  |  |  |
| **FQ - Emotionality** | .86** | .78** | .88** |  |  |  |  |  |
| **FQ - Affective Involvement** | .67** | .64** | .68** | .58** |  |  |  |  |
| **FQ - Control** | .63** | .58** | .64** | .67** | .56** |  |  |  |
| **FQ - Values and Norms** | .70** | .74** | .79** | .71** | .57** | .53** |  |  |
| **FQ - Social Expectation** | -.82** | -.84** | -.86** | -.85** | -.73** | -.68** | -.73** |  |
| **FQ - Defence** | -.70** | -.61** | -.68** | -.67** | -.49** | -.53** | -.50** | .57** |

*: *p* < .05, **: *p* < .01
