# Supplementary material for: Age Moderates the Relationships between Family Functioning and Neck Pain/Disability
Source: PLoS One. 2016 Apr 14;11(4):e0153606. doi: 10.1371/journal.pone.0153606 (PMC4831820; doi:10.1371/journal.pone.0153606)
Supplement: S2 Table — (DOCX) [file pone.0153606.s002.docx]

**S2 Table. Intercorrelations among the subscales of the Self-Estimating Questionnaire.**

|  | **SE - Task Accomplishment** | **SE - Role Performance** | **SE - Communication** | **SE - Emotionality** | **SE - Affective Involvement** | **SE - Control** |
| --- | --- | --- | --- | --- | --- | --- |
| **SE - Role Performance** | .66** |  |  |  |  |  |
| **SE - Communication** | .68** | .73** |  |  |  |  |
| **SE - Emotionality** | .69** | .66** | .80** |  |  |  |
| **SE - Affective Involvement** | .55** | .61** | .69** | .67** |  |  |
| **SE - Control** | .38** | .36** | .36** | .33** | .26* |  |
| **SE - Values and Norms** | .73** | .67** | .76** | .74** | .66** | .29** |

*: *p* < .05, **: *p* < .01
