# Supplementary material for: Age Moderates the Relationships between Family Functioning and Neck Pain/Disability
Source: PLoS One. 2016 Apr 14;11(4):e0153606. doi: 10.1371/journal.pone.0153606 (PMC4831820; doi:10.1371/journal.pone.0153606)
Supplement: S3 Table — (DOCX) [file pone.0153606.s003.docx]

**S3 Table. Intercorrelations among the subscales of the Diadic Relationship Scale.**

|  | **DR - Task Accomplishment** | **DR - Role Performance** | **DR - Communication** | **DR - Emotionality** | **DR - Affective Involvement** | **DR - Control** |
| --- | --- | --- | --- | --- | --- | --- |
| **DR - Role Performance** | .84** |  |  |  |  |  |
| **DR - Communication** | .87** | .85** |  |  |  |  |
| **DR - Emotionality** | .67** | .65** | .78** |  |  |  |
| **DR - Affective Involvement** | .75** | .79** | .84** | .85** |  |  |
| **DR - Control** | .74** | .75** | .80** | .76** | .82** |  |
| **DR - Values and Norms** | .79** | .85** | .83** | .75** | .87** | .83** |

*: *p* < .05, **: *p* < .01
