# Supplementary material for: Age Moderates the Relationships between Family Functioning and Neck Pain/Disability
Source: PLoS One. 2016 Apr 14;11(4):e0153606. doi: 10.1371/journal.pone.0153606 (PMC4831820; doi:10.1371/journal.pone.0153606)
Supplement: S4 Table — (DOCX) [file pone.0153606.s004.docx]

**S4 Table. Results of regression analyses testing for interactions between age and predictors (dependent variable: Neck Disability Index) - non-significant results.**

| **Predictor** | ***Beta*** | ***SE*** | ***t*** | ***p*** |
| --- | --- | --- | --- | --- |
| **FQ - Task Accomplishment** | 0.09 | 0.10 | 0.90 | .371 |
| **FQ - Control** | 0.10 | 0.10 | 1.09 | .281 |
| **FQ - Values and Norms** | 0.17 | 0.10 | 1.74 | .085 |
| **FQ - Defence** | -0.10 | 0.10 | -1.01 | .317 |
| **SE - Task Accomplishment** | 0.12 | 0.10 | 1.19 | .238 |
| **SE - Affective Involvement** | 0.19 | 0.12 | 1.60 | .113 |
| **SE - Values and Norms** | 0.19 | 0.10 | 1.91 | .059 |
| **DR - Role Performance** | 0.14 | 0.11 | 1.30 | .196 |
| **CISS - Task Oriented** | -0.04 | 0.11 | -0.39 | .699 |
| **CISS - Emotion Oriented** | 0.17 | 0.10 | 1.64 | .106 |
| **CISS - Avoidance Oriented** | 0.10 | 0.10 | 1.04 | .302 |
| **CISS - Involvement in other task** | 0.02 | 0.10 | 0.24 | .814 |
| **CISS - Social contacts** | 0.18 | 0.10 | 1.89 | .063 |
