# Supplementary material for: Age Moderates the Relationships between Family Functioning and Neck Pain/Disability
Source: PLoS One. 2016 Apr 14;11(4):e0153606. doi: 10.1371/journal.pone.0153606 (PMC4831820; doi:10.1371/journal.pone.0153606)
Supplement: S5 Table — (DOCX) [file pone.0153606.s005.docx]

**S5 Table. Results of regression analyses testing for interactions between age and predictors (dependent variable: Visual Analogue Scale (pain)) - non-significant results.**

| **Predictor** | ***Beta*** | ***SE*** | ***t*** | ***p*** |
| --- | --- | --- | --- | --- |
| **FQ - Task Accomplishment** | 0.09 | 0.12 | 0.76 | .451 |
| **FQ - Role Performance** | 0.17 | 0.11 | 1.53 | .131 |
| **FQ - Communication** | 0.09 | 0.12 | 0.74 | .460 |
| **FQ - Emotionality** | 0.13 | 0.11 | 1.17 | .244 |
| **FQ - Affective Involvement** | 0.20 | 0.11 | 1.85 | .068 |
| **FQ - Control** | 0.21 | 0.11 | 1.89 | .062 |
| **FQ - Social Expectation** | -0.20 | 0.11 | -1.80 | .076 |
| **FQ - Defence** | -0.17 | 0.11 | -1.55 | .126 |
| **SE - Task Accomplishment** | 0.19 | 0.11 | 1.66 | .101 |
| **SE - Role Performance** | 0.20 | 0.11 | 1.80 | .075 |
| **SE - Affective Involvement** | 0.04 | 0.14 | 0.26 | .799 |
| **SE - Control** | 0.15 | 0.12 | 1.26 | .213 |
| **SE - Values and Norms** | 0.19 | 0.12 | 1.56 | .123 |
| **DR - Task Accomplishment** | 0.19 | 0.12 | 1.54 | .128 |
| **DR - Role Performance** | 0.21 | 0.12 | 1.67 | .099 |
| **DR - Control** | 0.06 | 0.13 | 0.50 | .622 |
| **DR - Values and Norms** | 0.12 | 0.12 | 0.96 | .339 |
| **CISS - Task Oriented** | 0.01 | 0.13 | 0.05 | .959 |
| **CISS - Emotion Oriented** | 0.07 | 0.13 | 0.56 | .575 |
| **CISS - Avoidance Oriented** | 0.16 | 0.12 | 1.33 | .188 |
| **CISS - Involvement in other task** | 0.18 | 0.12 | 1.50 | .139 |
| **CISS - Social contacts** | 0.06 | 0.12 | 0.47 | .639 |
