# Supplementary material for: Age Moderates the Relationships between Family Functioning and Neck Pain/Disability
Source: PLoS One. 2016 Apr 14;11(4):e0153606. doi: 10.1371/journal.pone.0153606 (PMC4831820; doi:10.1371/journal.pone.0153606)
Supplement: S6 Table — (DOCX) [file pone.0153606.s006.docx]

**S6 Table. Multiple hierarchical-stepwise regressions for Neck Disability Index as the dependent variable and family functioning (Family Questionnaire) as predictors - non-significant results.**

| **Predictor** | ***Beta*** | ***t*** | ***p*** | ***Tolerance*** |
| --- | --- | --- | --- | --- |
| **FQ - Role Performance** | 0.00 | 0.02 | .985 | 0.33 |
| **FQ - Communication** | 0.04 | 0.26 | .799 | 0.34 |
| **FQ - Emotionality** | -0.07 | -0.39 | .698 | 0.28 |
| **FQ - Affective Involvement** | 0.12 | 1.03 | .305 | 0.65 |
| **FQ - Control** | -0.09 | -0.73 | .467 | 0.59 |
| **FQ - Values and Norms** | 0.07 | 0.55 | .581 | 0.57 |
| **FQ - Social Expectation** | -0.13 | -0.83 | .409 | 0.38 |
| **FQ - Defence** | 0.06 | 0.52 | .606 | 0.61 |
