# Supplementary material for: Age Moderates the Relationships between Family Functioning and Neck Pain/Disability
Source: PLoS One. 2016 Apr 14;11(4):e0153606. doi: 10.1371/journal.pone.0153606 (PMC4831820; doi:10.1371/journal.pone.0153606)
Supplement: S7 Table — (DOCX) [file pone.0153606.s007.docx]

**S7 Table. Multiple hierarchical-stepwise regressions for Neck Disability Index as the dependent variable and family functioning (Self-Estimating Questionnaire) as predictors - non-significant results.**

| **Predictor** | ***Beta*** | ***t*** | ***p*** | ***Tolerance*** |
| --- | --- | --- | --- | --- |
| **SE - Task Accomplishment** | 0.06 | 0.55 | .588 | 0.90 |
| **SE - Role Performance** | 0.09 | 0.93 | .357 | 0.98 |
| **SE - Communication** | 0.17 | 1.75 | .084 | 0.98 |
| **SE - Emotionality** | 0.12 | 1.14 | .259 | 0.94 |
| **SE - Affective Involvement** | 0.19 | 1.94 | .056 | 0.95 |
| **SE - Control** | 0.14 | 1.40 | .164 | 0.98 |
| **SE - Values and Norms** | 0.19 | 1.84 | .069 | 0.93 |
