# Supplementary material for: Age Moderates the Relationships between Family Functioning and Neck Pain/Disability
Source: PLoS One. 2016 Apr 14;11(4):e0153606. doi: 10.1371/journal.pone.0153606 (PMC4831820; doi:10.1371/journal.pone.0153606)
Supplement: S8 Table — (DOCX) [file pone.0153606.s008.docx]

**S8 Table. Multiple hierarchical-stepwise regressions for Neck Disability Index as the dependent variable and family functioning (Diadic Relationship Scale) as predictors - non-significant results.**

| **Predictor** | ***Beta*** | ***t*** | ***p*** | ***Tolerance*** |
| --- | --- | --- | --- | --- |
| **DR - Task Accomplishment** | 0.16 | 1.58 | .118 | 0.98 |
| **DR - Role Performance** | 0.10 | 0.93 | .357 | 0.99 |
| **DR - Communication** | 0.10 | 0.92 | .363 | 1.00 |
| **DR - Emotionality** | 0.11 | 1.07 | .290 | 0.98 |
| **DR - Affective Involvement** | 0.09 | 0.87 | .386 | 0.97 |
| **DR - Control** | 0.04 | 0.34 | .734 | 0.99 |
| **DR - Values and Norms** | 0.10 | 0.92 | .361 | 0.98 |
| **DR - Task Accomplishment** | 0.16 | 1.58 | .118 | 0.98 |
| **DR - Role Performance** | 0.10 | 0.93 | .357 | 0.99 |
