# Supplementary material for: Age Moderates the Relationships between Family Functioning and Neck Pain/Disability
Source: PLoS One. 2016 Apr 14;11(4):e0153606. doi: 10.1371/journal.pone.0153606 (PMC4831820; doi:10.1371/journal.pone.0153606)
Supplement: S9 Table — (DOCX) [file pone.0153606.s009.docx]

**S9 Table. Multiple hierarchical-stepwise regressions for Neck Disability Index as the dependent variable and coping styles as predictors - non-significant results.**

| **Predictor** | ***Beta*** | ***t*** | ***p*** | ***Tolerance*** |
| --- | --- | --- | --- | --- |
| **CISS - Task Oriented** | 0.03 | 0.30 | .767 | 0.90 |
| **CISS - Emotion Oriented** | -0.08 | -0.71 | .481 | 0.92 |
| **CISS - Avoidance Oriented** | -0.15 | -1.45 | .151 | 1.00 |
| **CISS - Involvement in other task** | -0.14 | -1.33 | .188 | 0.98 |
| **CISS - Social contacts** | -0.06 | -0.51 | .611 | 0.91 |
