# Supplementary material for: Age Moderates the Relationships between Family Functioning and Neck Pain/Disability
Source: PLoS One. 2016 Apr 14;11(4):e0153606. doi: 10.1371/journal.pone.0153606 (PMC4831820; doi:10.1371/journal.pone.0153606)
Supplement: S10 Table — (DOCX) [file pone.0153606.s010.docx]

**S10 Table. Multiple hierarchical-stepwise regressions for the Visual-Analogue Scale (pain) as the dependent variable and family functioning (Family Questionnaire) as predictors - non-significant results.**

| **Predictor** | ***Beta*** | ***t*** | ***p*** | ***Tolerance*** |
| --- | --- | --- | --- | --- |
| **FQ - Task Accomplishment** | 0.25 | 1.91 | .060 | 0.65 |
| **FQ - Role Performance** | 0.24 | 1.93 | .058 | 0.71 |
| **FQ - Communication** | 0.22 | 1.63 | .108 | 0.63 |
| **FQ - Emotionality** | 0.15 | 1.13 | .264 | 0.69 |
| **FQ - Control** | -0.18 | -1.36 | .177 | 0.65 |
| **FQ - Values and Norms** | 0.02 | 0.12 | .905 | 0.71 |
| **FQ - Social Expectation** | -0.14 | -0.94 | .353 | 0.53 |
| **FQ - Defence** | -0.16 | -1.33 | .188 | 0.85 |
