# Supplementary material for: Age Moderates the Relationships between Family Functioning and Neck Pain/Disability
Source: PLoS One. 2016 Apr 14;11(4):e0153606. doi: 10.1371/journal.pone.0153606 (PMC4831820; doi:10.1371/journal.pone.0153606)
Supplement: S11 Table — (DOCX) [file pone.0153606.s011.docx]

**S11 Table. Multiple hierarchical-stepwise regressions for Visual-Analogue Scale (pain) as the dependent variable and family functioning (Self-Estimating Questionnaire) as predictors - non-significant results.**

| **Predictor** | ***Beta*** | ***t*** | ***p*** | ***Tolerance*** |
| --- | --- | --- | --- | --- |
| **SE - Task Accomplishment** | 0.03 | 0.16 | .871 | 0.47 |
| **SE - Role Performance** | 0.05 | 0.31 | .758 | 0.47 |
| **SE - Emotionality** | 0.03 | 0.16 | .873 | 0.34 |
| **SE - Affective Involvement** | 0.17 | 1.06 | .293 | 0.49 |
| **SE - Control** | -0.04 | -0.34 | .736 | 0.82 |
| **SE - Values and Norms** | -0.06 | -0.34 | .736 | 0.36 |
