# Supplementary material for: Age Moderates the Relationships between Family Functioning and Neck Pain/Disability
Source: PLoS One. 2016 Apr 14;11(4):e0153606. doi: 10.1371/journal.pone.0153606 (PMC4831820; doi:10.1371/journal.pone.0153606)
Supplement: S12 Table — (DOCX) [file pone.0153606.s012.docx]

**S12 Table. Multiple hierarchical-stepwise regressions for Visual-Analogue Scale (pain) as the dependent variable and family functioning (Diadic Relationship Scale) as predictors - non-significant results.**

| **Predictor** | ***Beta*** | ***t*** | ***p*** | ***Tolerance*** |
| --- | --- | --- | --- | --- |
| **DR - Task Accomplishment** | 0.04 | 0.18 | .854 | 0.36 |
| **DR - Role Performance** | 0.06 | 0.23 | .819 | 0.23 |
| **DR - Communication** | -0.19 | -0.85 | .399 | 0.29 |
| **DR - Emotionality** | -0.28 | -1.47 | .147 | 0.39 |
| **DR - Affective Involvement** | -0.39 | -1.49 | .142 | 0.21 |
| **DR - Control** | -0.06 | -0.25 | .802 | 0.27 |
