# Supplementary material for: Age Moderates the Relationships between Family Functioning and Neck Pain/Disability
Source: PLoS One. 2016 Apr 14;11(4):e0153606. doi: 10.1371/journal.pone.0153606 (PMC4831820; doi:10.1371/journal.pone.0153606)
Supplement: S13 Table — (DOCX) [file pone.0153606.s013.docx]

**S13 Table. Multiple hierarchical-stepwise regressions for Visual-Analogue Scale (pain) as the dependent variable and coping styles (CISS) as predictors - non-significant results.**

| **Predictor** | ***Beta*** | ***t*** | ***p*** | ***Tolerance*** |
| --- | --- | --- | --- | --- |
| **CISS - Task Oriented** | 0.15 | 1.09 | .279 | 0.90 |
| **CISS - Emotion Oriented** | 0.02 | 0.15 | .881 | 0.92 |
| **CISS - Avoidance Oriented** | 0.02 | 0.15 | .885 | 0.99 |
| **CISS - Involvement in other task** | 0.02 | 0.14 | .887 | 0.98 |
| **CISS - Social contacts** | 0.01 | 0.04 | .965 | 0.90 |
